# Little Known Dangers of an Exotic Poisonous Fruit: Lessons From Two Cases of Konjac Ingestion

**DOI:** 10.7759/cureus.11972

**Published:** 2020-12-08

**Authors:** Roshni Pillay, Faiz Mukthar Chemban, Vijay V Pillay, Balram Rathish

**Affiliations:** 1 Poison Control Centre, Amrita Institute of Medical Sciences, Kochi, IND; 2 Internal Medicine, Taluk Hospital Malappuram, Malappuram, IND; 3 Infectious Diseases, Aster Medcity, Kochi, IND

**Keywords:** amorphophallus konjac, konjac, poisoning, kerala

## Abstract

*Amorphophallus konjac*, often abbreviated to Konjac, is a perennial plant that is not uncommon in parts of Asia where it is cultivated as a food source. However, consumption of the raw plant has been known to be toxic to animals. We report the first human cases of Konjac poisoning in two children after accidental ingestion of its seeds.

## Introduction

*Amorphophallus konjac* which is known by common names such as Devil's tongue, Voodoo lily, or quite simply Konjac, is a perennial plant that is cultivated as food in parts of China and Japan, but is also found in other parts of Asia [1,2]. The plant has a corm (a tuber-like structure) which is edible once it is dried and cooked, but is toxic when consumed raw, due to the presence of calcium oxalate crystals in it [1]. The other parts of the plant too are thought to contain the same crystals, that cause intense irritation to the mucosa of the oral cavity and esophagus. Cases of such toxicity have been reported in cats and dogs [2].

An extensive review of the literature revealed that human cases of direct Konjac poisoning have not been reported, though there is one case of gastroparesis and persistent vomiting in an Australian woman following the consumption of noodles made from Konjac flour [3]. Here, we present the case of two sibling children with ingestion of konjac seeds, and the clinical outcome in both.

## Case presentation

Case 1

**Figure 1:**
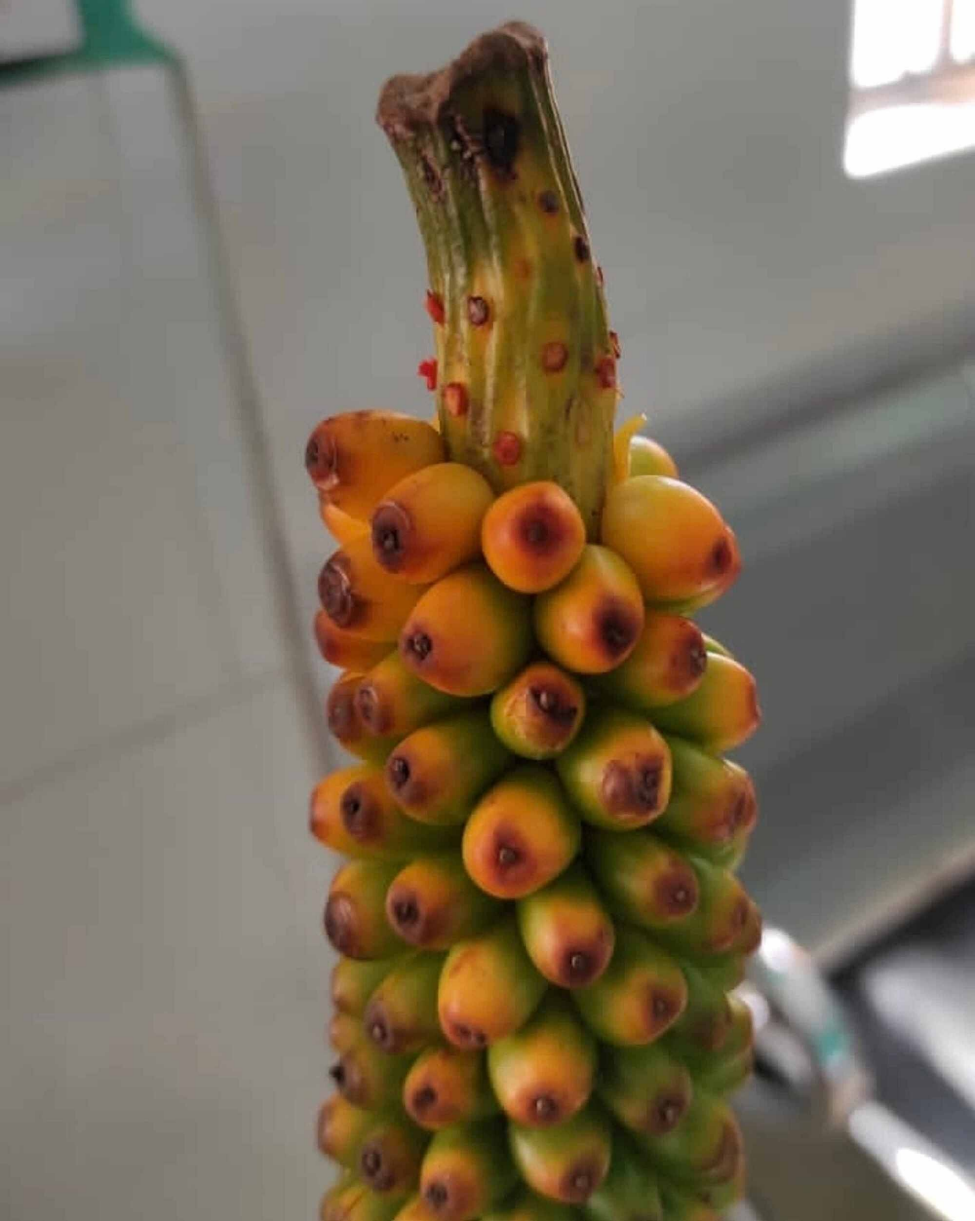
Amorphophallus konjac seeds

A four-year-old boy from the northern region of the state of Kerala in India was presented with multiple episodes of vomiting immediately after ingesting two seeds of a plant while playing with his sibling (Figure 1). He did not have any significant past medical history. Clinical examination revealed an alert child with some erythema over the tongue and oral mucosa, and features of possible dehydration. The systemic examination was normal. Gastric lavage was not attempted as more than two hours had elapsed following ingestion, and also because of suspicion of irritant nature of the toxin. The plant was later identified to be *A. konjac* and the child was given an intravenous fluid replacement and observed for about 12 hours. But at the parents' insistence, he was discharged after that with regular telephonic follow up. The child was reported to be active with complete resolution of the erythema, about a day later, over a telephonic review.

Case 2

**Figure 2:**
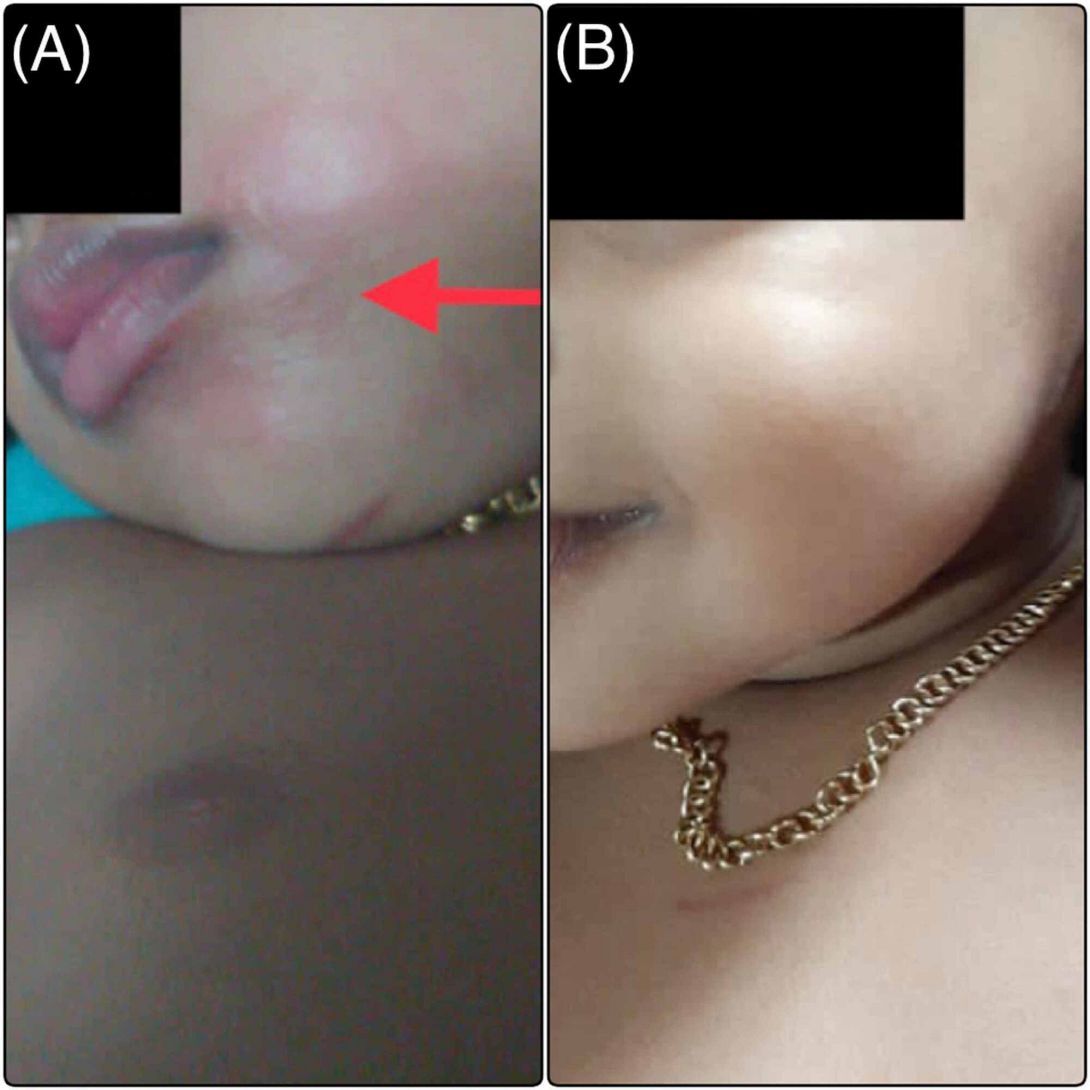
(A) Erythematous rash seen around the oral cavity of the child (red arrow). (B) Complete resolution of the rash after 24 hours.

A two-year-old girl, who was the younger sister of case 1, was presented with multiple episodes of vomiting immediately following the ingestion of an unknown number of seeds of the same plant while playing. She too did not have any significant past medical history. Clinical examination showed an alert child with an erythematous macular rash surrounding the mouth, as well as erythema over the tongue and oral mucosa (Figure 2A). There were signs of dehydration. The systemic examination was normal. Gastric lavage was not attempted for the same reasons as in the case of her sibling. The child was given an intravenous fluid replacement and observed for about 12 hours, but just as in the case of her sibling, on the parents' insistence, was discharged after that with regular telephonic follow up. She too was reported to be active with complete resolution of the erythema and rash 24 hours later, over the telephonic review (Figure 2B).

## Discussion

These two cases represent possibly the first-ever reported human cases of *A. konjac* poisoning in the medical literature. Even though it is known that Konjac fruit contains calcium oxalate crystals and is an irritant if consumed raw, the systemic effects of ingestion are not well understood [1]. In our cases, both children had only mild to moderate vomiting with some extent of dehydration. While one child had an erythematous reaction of the skin, both had erythema of the tongue and oral mucosa. There were no systemic manifestations identified in either child in the first 12 hours after ingestion and on the 24-hour telephonic review. 

The only other case reported in the medical literature involving Konjac concerned a 61-year-old Australian woman who had consumed packaged noodles made from Konjac flour. She presented with gastric outlet obstruction and persistent vomiting. However, in that case, the manifestations were a result of a vegetable bezoar forming in the stomach of the victim with subsequent gastric outlet obstruction, and not due to the inherent toxicity of Konjac, as the flour is dried and cooked before consumption [3].

Pillay and Olsen et al. suggest that for cases of oxalic acid and calcium oxalate poisoning, administration of calcium gluconate should be considered, as the absorption of oxalate can cause acute hypocalcemia secondary to precipitation of the insoluble calcium oxalate salts [4,5]. However, Olsen et al. are of the opinion that the insoluble calcium oxalate salts which are found in plants such as Dieffenbachia, as well as similar other plants, may not be absorbed, and usually, only cause a local reaction in the gastro-intestinal mucous membrane [5]. This may be the case with regard to Konjac as well.

Unfortunately, neither of our patients underwent body fluid investigations (blood, urine, gastric aspirate) and we were unable to admit both children for continuous observation in view of the current COVID-19 pandemic and the understandable anxiety of the parents to avoid prolonged exposure of their children to a hospital environment. Despite these limitations, our cases substantiate the fact that Konjac may only cause local manifestations in the gastrointestinal tract, and systemic effects may be negligible, based on the findings from these cases.

## Conclusions

Konjac is a plant that is found in certain parts of Asia which has a potential for accidental poisoning in children in view of its attractive appearance. Konjac poisoning presents predominantly as vomiting due to the irritation of the oral and gastric mucosa by the calcium oxalate crystals found in all parts of the plant. The systemic effects of raw Konjac consumption are not clear as there have not been any reported human cases until our cases.

The conservative management approach adopted in our cases with the subsequent complete recovery made by both children suggests that Konjac poisoning may not be life-threatening, but only has manifestations due to its irritant nature. However, further data are required before this can be conclusively stated as the clinical manifestations and severity may vary depending on the quantity as well as part of the plant that is ingested.
